# Fibromatosis-Like Metaplastic Triple-Negative Breast Cancer: A Case Report

**DOI:** 10.7759/cureus.114224

**Published:** 2026-08-09

**Authors:** Milo J Cohen, Alena Padilla Rocca, Nicole J Nelles, Kiran Chang

**Affiliations:** 1 General Medicine, The Kinkaid School, Houston, USA; 2 College of Natural and Health Sciences, University of Tampa, Tampa, USA; 3 Department of Pathology and Genomic Medicine, Houston Methodist Hospital, Houston, USA; 4 Department of Diagnostic and Interventional Imaging, The University of Texas Health Science Center at Houston, Houston, USA

**Keywords:** breast conservation, fibromatosis-like metaplastic carcinoma, metaplastic breast carcinoma, spindle cell lesion, triple-negative breast cancer

## Abstract

Fibromatosis-like metaplastic carcinoma (FLMC) is an extremely rare subtype of metaplastic breast carcinoma that closely resembles desmoid-type fibromatosis histologically, making it one of the most diagnostically challenging breast lesions. In contrast to most triple-negative breast cancers, FLMC follows a relatively indolent clinical course, though local recurrence is well documented, and because so few cases have been reported, no established treatment guidelines exist and the role of chemotherapy remains uncertain. We present the case of a 63-year-old woman recalled from routine screening digital breast tomosynthesis for an irregular, spiculated mass in the right breast, confirmed on biopsy to be FLMC, and treated with breast-conserving surgery and adjuvant radiation therapy without chemotherapy after two medical oncologists gave opposing recommendations regarding systemic treatment. She has remained without evidence of disease at two-year follow-up. This case adds to the limited literature on FLMC, supports surgery and radiation alone as a potentially effective treatment strategy in carefully selected patients, and highlights the importance of recognizing FLMC as a biologically distinct entity that should not be managed the same way as conventional triple-negative breast cancer, though longer-term follow-up is needed given the limited data on treatment outcomes for this rare tumor.

## Introduction

Metaplastic breast carcinoma (MBC) is a rare group of breast cancers, making up less than 0.5% of all breast cancer diagnoses [1]. In these tumors, the typical glandular epithelium is replaced, either partly or fully, by other cell types, including squamous, spindle, or mesenchymal elements. The term metaplastic refers to this process in which tumor cells take on the appearance of tissue types not normally found in the breast, such as spindle or squamous cells, rather than resembling typical breast glandular epithelium. Nearly all cases are triple-negative, meaning they lack estrogen receptor (ER), progesterone receptor (PR), and human epidermal growth factor receptor 2 (HER2) expression. The triple-negative immunophenotype is clinically significant because it precludes the use of endocrine therapy and HER2-targeted agents, leaving cytotoxic chemotherapy as the only systemic treatment option for conventional triple-negative breast cancer. Fibromatosis-like metaplastic carcinoma (FLMC) is an especially rare subtype within this group. Histologically, FLMC closely resembles desmoid-type fibromatosis, a benign fibroblastic proliferation, which predisposes FLMC to misdiagnosis, particularly on core needle biopsy where tissue sampling may be limited [1,2], and is an important distinction given that desmoid fibromatosis does not metastasize but can locally infiltrate and recur, whereas FLMC is a true epithelial malignancy that must be recognized and treated as such.

Despite being a true malignancy, FLMC follows a generally indolent clinical course relative to conventional triple-negative and MBC; it rarely spreads to the regional lymph nodes or distant organs, though local recurrence and rare distant metastases are well documented [3]. This has raised the question of whether chemotherapy, a standard component of treatment for most triple-negative breast cancers, is necessary for FLMC. FLMC was first described as a distinct entity by Gobbi et al. in 1999 [4], and because this triple-negative subtype is so rare, no large clinical trials exist; treatment decisions are based mainly on individual case reports and small patient series. Fewer than 100 cases have been reported in the medical literature over the past 20 years [5,6].

Herein, we present a case of FLMC in a woman treated with breast-conserving surgery and radiation therapy, without chemotherapy. Our goal is to add to the limited body of literature on this tumor and to highlight its key imaging, pathologic, and clinical features.

## Case presentation

**Figure 1:**
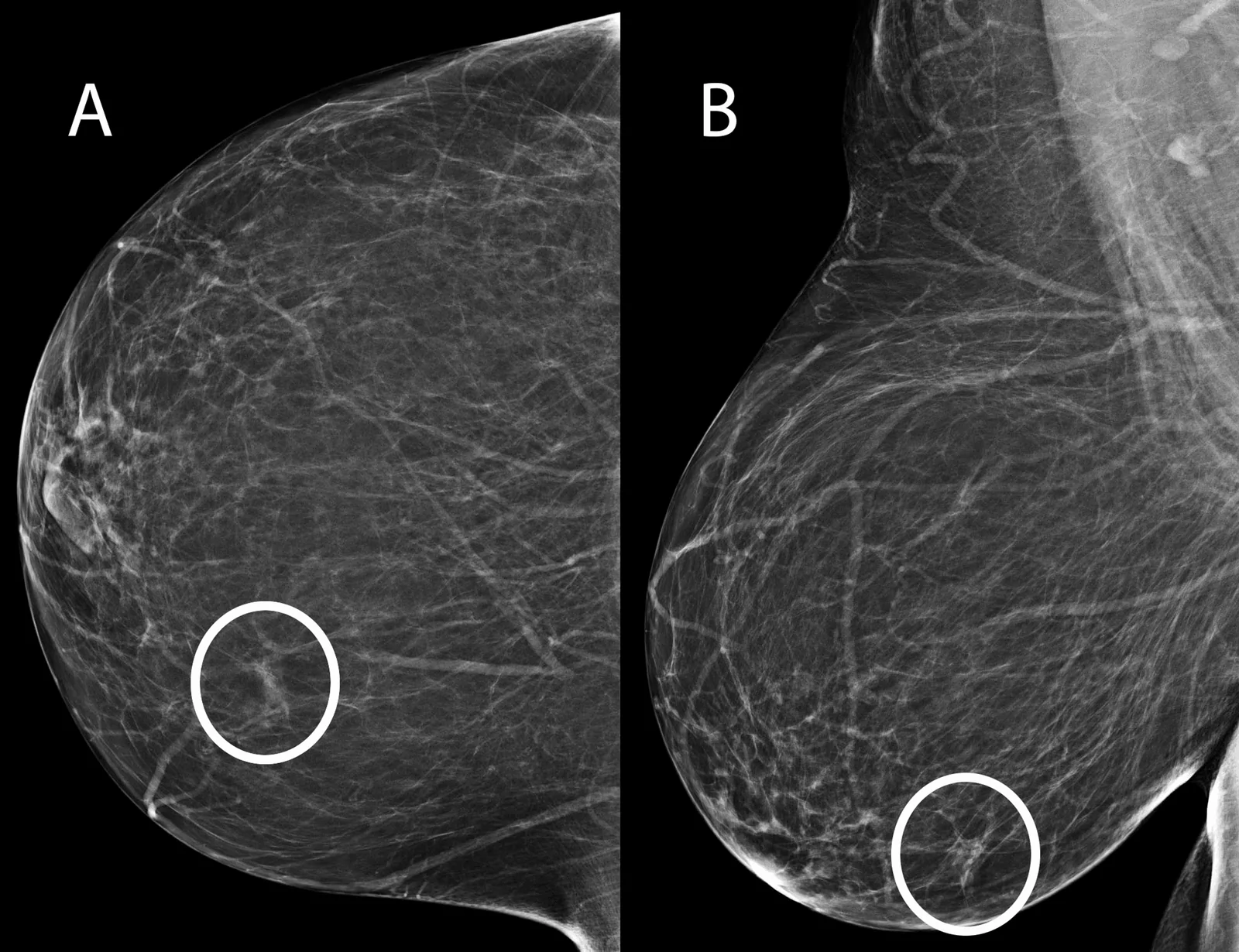
Initial screening mammogram. (A) Craniocaudal and (B) mediolateral oblique digital mammographic views of the right breast for screening demonstrate an abnormal focal asymmetry (open white circles) in the lower inner quadrant. Breast Imaging Reporting and Data System (BI-RADS) category: 0 - incomplete: needs additional imaging evaluation.

**Figure 2:**
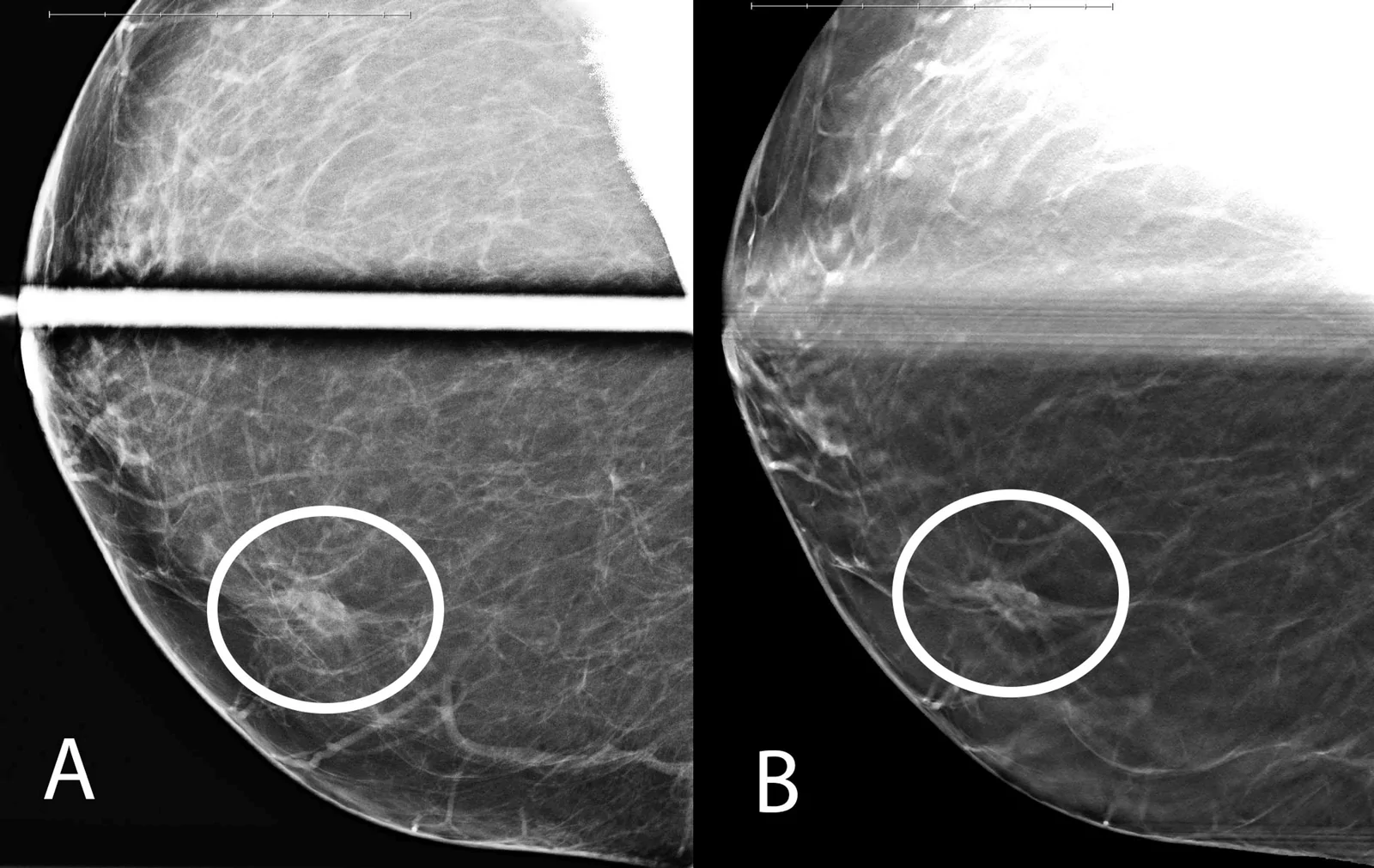
Diagnostic mammogram following the BI-RADS assessment category 0 screening mammogram. (A) Spot compression craniocaudal view and (B) spot compression tomosynthesis craniocaudal image reveal persistence of the focal asymmetry (open white circles) and associated architectural distortion. This finding was new compared with prior mammograms and was assigned a Breast Imaging Reporting and Data System (BI-RADS) category 4. The architectural distortion was not appreciable on the initial screening mammogram and was only identified on spot compression tomosynthesis views.

**Figure 3:**
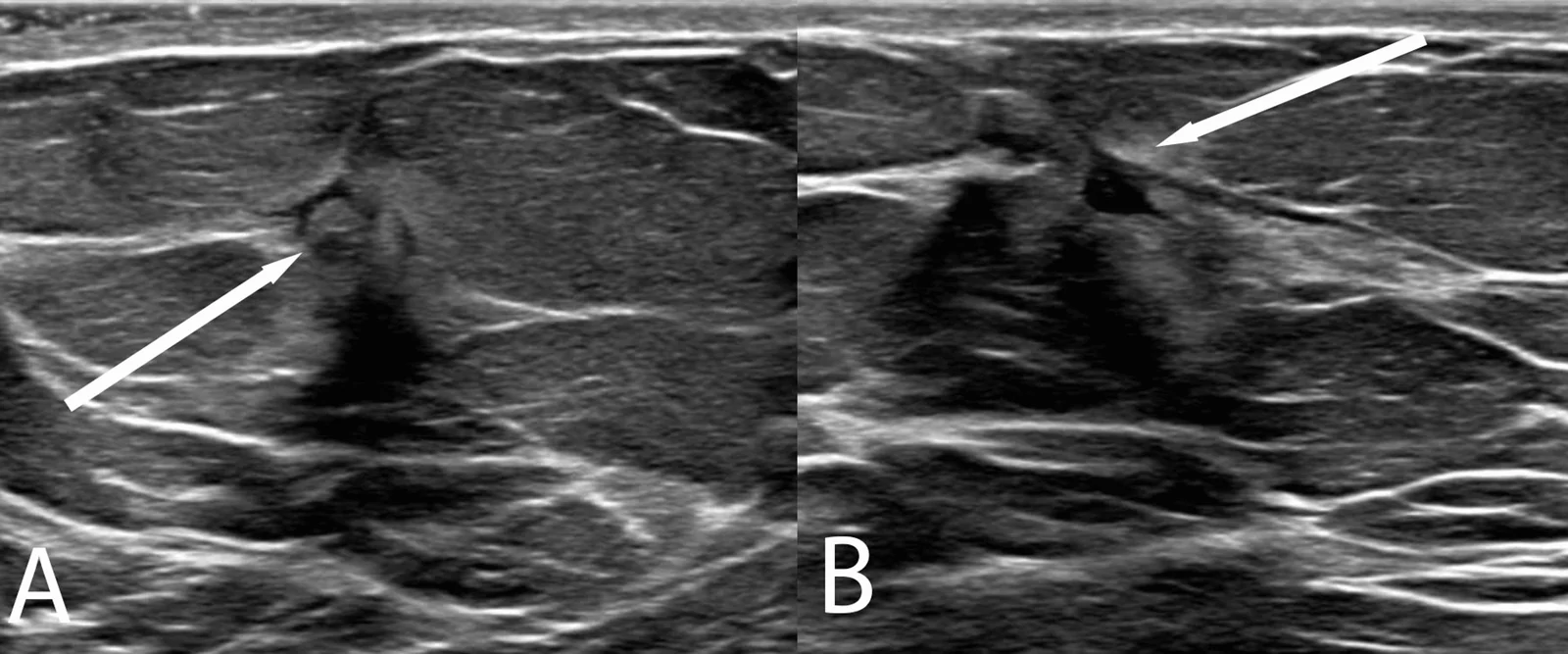
Targeted ultrasound of the right breast performed on the same day as the diagnostic mammogram. (A) Longitudinal and (B) transverse images of the right breast at 4:00 demonstrate an irregularly shaped, mixed-echogenicity mass with angular margins measuring 1.8 cm (white arrows). This was classified as suspicious and given a Breast Imaging Reporting and Data System (BI-RADS) assessment category 4 on ultrasound. Ultrasound-guided biopsy was subsequently recommended.

A 63-year-old postmenopausal woman was recalled from routine screening mammography with digital breast tomosynthesis for further evaluation of a newly detected focal asymmetry in the right breast (Figure 1). She was asymptomatic, with no palpable mass, nipple discharge, or skin changes, and had no prior abnormal mammograms. Relevant medical history included depression, psoriasis, medically controlled hypertension, and hypercholesterolemia. She had used a combined estrogen-progestogen transdermal hormone replacement patch for an extended period prior to diagnosis, though the exact duration was unknown. Family history was notable for breast cancer in a maternal grandmother diagnosed in her 90s. Diagnostic mammography with tomosynthesis two weeks later confirmed persistence of a 1.6 cm focal asymmetry in the right lower inner quadrant (Figure 2). Targeted ultrasound demonstrated a corresponding irregular mixed echogenicity mass measuring 1.8 cm with angular margins and posterior acoustic shadowing (Figure 3). It was classified as Breast Imaging Reporting and Data System (BI-RADS) assessment category 4, suspicious. There was no associated regional lymphadenopathy. Ultrasound-guided core needle biopsy was recommended and subsequently performed.

**Figure 4:**
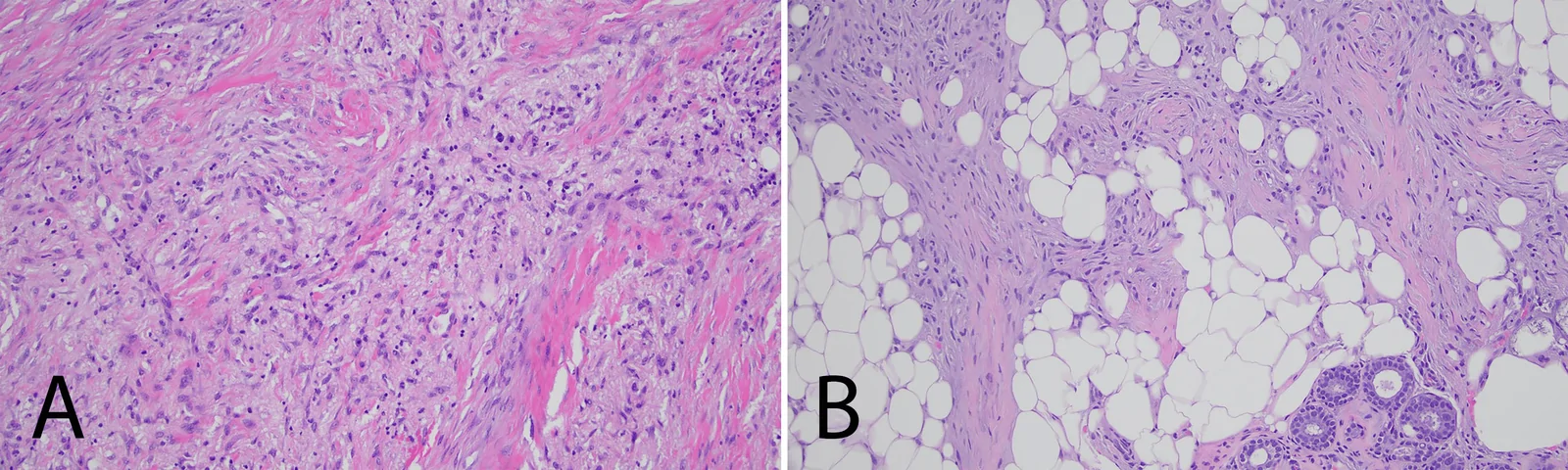
Histopathology (A) H&E staining from the partial mastectomy specimen at 20X objective magnification shows that the tumor is composed of bland spindled cells with pale eosinophilic cytoplasm in wavy fascicles and short cords with a collagenous background. (B) H&E staining from the partial mastectomy specimen at 20X objective magnification also shows that the tumor infiltrated the fat.

**Figure 5:**
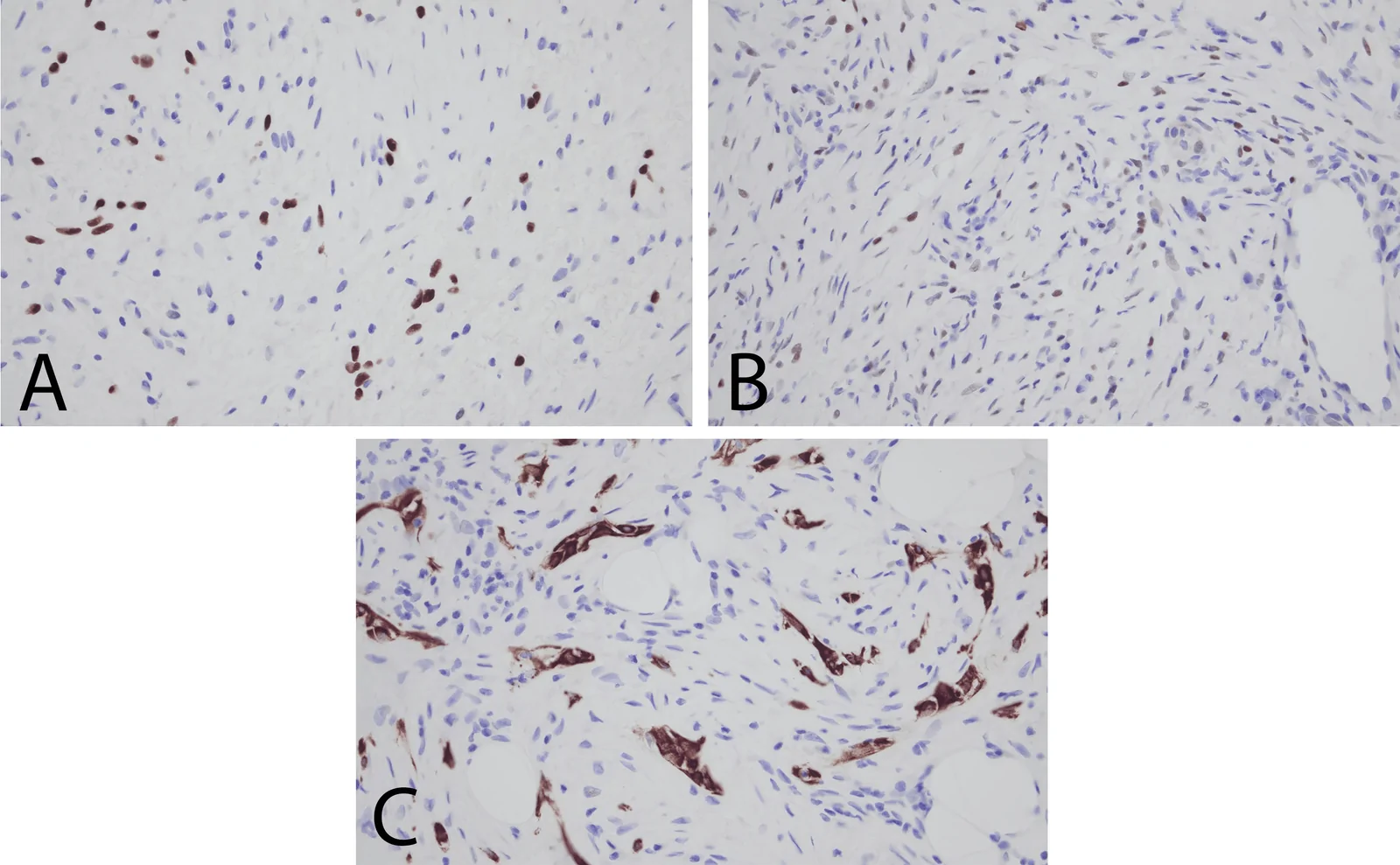
Immunohistochemical stains (A) The p63 immunostain highlights the nuclei of the tumor cells in brown, confirming carcinoma origin. (B) GATA-3 immunostain shows positive weak expression of the nuclei, supporting a carcinoma of breast origin. (C) Cytokeratin cocktail immunostain stains the cytoplasm brown of the spindled neoplastic cells, confirming this is a carcinoma instead of a mesenchymal tumor (i.e., sarcoma). Note that no single immunostain independently confirms a diagnosis of fibromatosis-like metaplastic carcinoma (FLMC); interpretation requires correlation with the complete morphologic and immunohistochemical profile.

**Figure 6:**
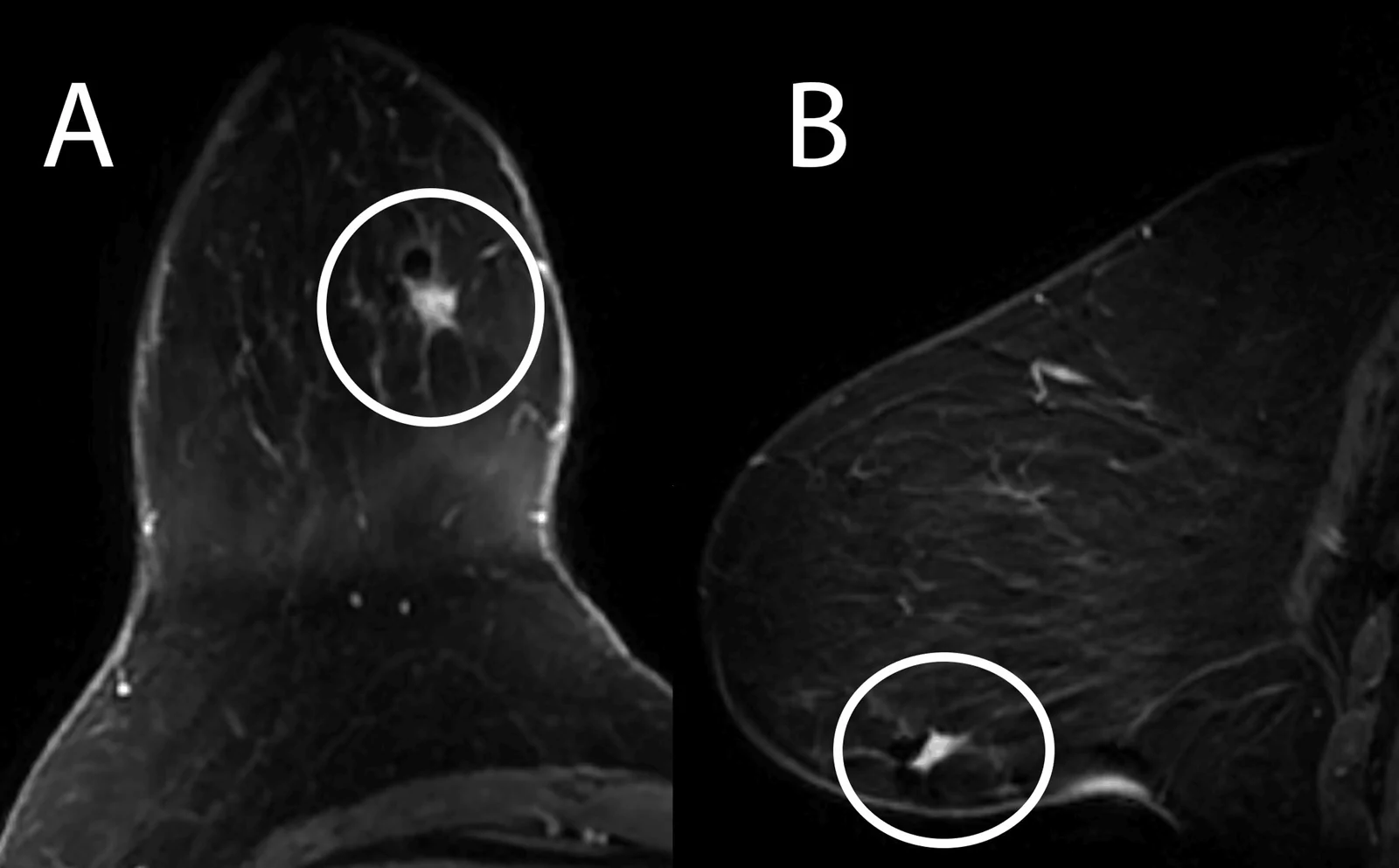
Preoperative breast MRI performed for extent of disease. (A) Axial and (B) sagittal MR images of the right breast, after percutaneous biopsy diagnosed fibromatosis-like metaplastic triple negative breast cancer, redemonstrate the malignancy as an irregular mass with spiculations (open white circle). There was no evidence of multicentric disease, multifocal disease, or contralateral synchronous malignancy.

Histologically, the tumor was composed of bland spindled cells with pale eosinophilic cytoplasm in wavy fascicles and short cords with a collagenous background (Figures 4, 5). No prominent nuclear atypia, increased mitotic activity, or necrosis was seen. No background in situ carcinoma was seen in the adjacent breast tissue. Immunohistochemical staining demonstrates the spindled tumor cells were positive for the following stains: cytokeratin cocktail, CK903, CK8/18, CK5/6, CK7, p63, p40, GATA3, SM-actin, and calponin. The tumor cells were negative for EMA, SOX10, S100, ERG, desmin, SM-myosin, CD34, beta-catenin, STAT6, MUC4, and ALK-4. This immunoprofile with evidence of epithelial differentiation by a variety of keratin stains was supportive of a diagnosis of FLMC. It is important to note that extensive tissue sampling is essential prior to rendering a definitive diagnosis of FLMC, as mixed metaplastic elements including squamous or glandular components may be present in a subset of cases and would not be apparent on limited core biopsy sampling. Identification of such elements can alter the tumor classification and may have implications for prognosis and management. The tumor was ER negative (0% tumor cells positive), PR negative (0% tumor cells positive), negative for HER2 by immunohistochemistry (IHC) (ASCO/CAP score 0), and demonstrated a low Ki-67 proliferation rate (12%). Clinical stage was cT1c N0 M0, stage IIA. A staging preoperative breast MRI (Figure 6) revealed no evidence of multicentric or multifocal disease. 

The patient underwent a partial mastectomy following Savi Scout localization three months after her diagnosis, which showed a 26 mm tumor that was completely excised with negative margins. No lymphovascular invasion or perineural invasion was identified. The overlying skin was uninvolved by tumor. Three sentinel lymph nodes were biopsied which were negative for metastatic carcinoma. Final pathologic staging was pT2 N0 M0, stage IIA.

Regarding systemic therapy, the patient was evaluated by two community-based medical oncologists from separate medical practices with opposite recommendations. The first oncologist, treating this as standard triple-negative metaplastic disease, recommended adjuvant chemotherapy with either dose-dense doxorubicin and cyclophosphamide followed by paclitaxel or docetaxel and cyclophosphamide with granulocyte colony-stimulating factor support. The second oncologist, acknowledging the fibromatosis-like histologic subtype and its recognized indolent biology with low metastatic potential, recommended against systemic chemotherapy. After a thorough discussion of the available evidence and the uncertainty surrounding this rare tumor, the patient chose to decline chemotherapy. The patient subsequently completed adjuvant whole-breast radiation therapy without complication. She has remained without evidence of disease after two years of clinical examinations and routine annual follow-up mammography and breast MRI.

## Discussion

FLMC is an extremely rare subtype of metaplastic, triple-negative breast cancer, and most of what is known about it comes from individual case reports and small patient series. A review of the English-language literature identified approximately 70 reported cases prior to 2021, with outcome data available for 41; among these, local recurrence occurred in 14 cases, regional lymph node involvement in only three, and distant metastases in five [4-6]. Diagnosing it correctly remains a significant challenge because its bland spindle cell morphology and collagenous stroma appear similar histologically to several benign and low-grade lesions, most notably desmoid-type fibromatosis [7]. It is important to note that imaging findings in FLMC are not pathognomonic for this specific subtype; a suspicious mass on mammography or ultrasound can prompt biopsy but cannot establish the diagnosis. A definitive diagnosis requires histologic examination, a comprehensive immunohistochemical panel, and careful exclusion of desmoid fibromatosis and other spindle cell lesions. Misdiagnosis is problematic because categorizing it as a benign tumor, most often desmoid-type fibromatosis, can lead to inadequate surgical excision and delays in appropriate treatment. The diagnostic challenge is further compounded with core needle biopsy, where limited sampling may yield only bland collagenous stroma without representative spindle cells or interpretable IHC markers. In such cases, repeat sampling or excision might be necessary to establish the correct diagnosis [8,9].

Histopathology and differential diagnosis

The defining histologic feature of FLMC is a proliferation of bland spindle cells with minimal nuclear atypia and low mitotic activity, arranged within a collagen-rich stromal background. This morphology closely resembles desmoid-type fibromatosis. Because the typical cytoarchitectural features of carcinoma are absent, IHC staining is necessary to confirm epithelial differentiation [5,10]. The IHC profile of FLMC is fairly consistent: stains for cytokeratins (especially high-molecular-weight types) and p63 are positive, while ER, PR, HER2, and CD34 are negative [4,10,11]. The most helpful stain for distinguishing FLMC and desmoid-type fibromatosis is β-catenin. In desmoid fibromatosis, β-catenin accumulates within the cell nucleus because of mutations in the CTNNB1 or APC genes [12]. In FLMC, this nuclear buildup does not occur; instead, β-catenin staining is seen only in the cell membrane and cytoplasm [5,10,12]. Other entities in the differential diagnosis include myofibroblastoma, inflammatory myofibroblastic tumor, pseudoangiomatous stromal hyperplasia, and adenomyoepithelioma, all of which can be reliably distinguished with a comprehensive IHC panel and thorough histologic review.

Clinical behavior and prognosis

Although FLMC carries a triple-negative immunophenotype, a feature typically associated with aggressive biological behavior, it follows a considerably more indolent clinical course than other triple-negative cancers. Regional lymph node and distant metastases are uncommon, though local recurrence remains a well-recognized risk [7]. Still, it would be a mistake to assume that all FLMC cases are harmless. Rare examples of aggressive behavior have been reported. In one recent case, lung metastasis appeared just five months after surgery, followed by bone metastasis at 15 months, despite treatment with surgery, radiation, and multiple rounds of chemotherapy [1]. Genetic testing in that case found a TERT promoter mutation. Importantly, TERT promoter mutation has since been identified as a recurrent molecular feature of FLMC more broadly, not an isolated finding limited to aggressive cases [11]. These molecular findings support FLMC as a biologically distinct tumor subtype within low-grade metaplastic breast cancer. However, available data are insufficient to establish TERT promoter mutation as an independent prognostic biomarker, and its clinical significance in risk stratification remains to be determined.

Management considerations

Since FLMC is so uncommon, there are no established treatment guidelines based on large clinical trials. Treatment decisions have largely been based off of small case series and what is known about desmoid-type fibromatosis. Surgery to excise the malignancy with clear margins is the principal treatment. Because FLMC so rarely spreads to regional lymph nodes, complete axillary lymph node dissection is generally not recommended [2,5,13]. The roles of radiation therapy and chemotherapy after surgery are not well established. Some authors have suggested that radiation may lower the risk of local recurrence, especially for bigger tumors, but this has not been confirmed in controlled studies [2,4,5,12,13].

The treatment approach in our patient, consisting of lumpectomy followed by adjuvant radiation without chemotherapy, is consistent with current expert recommendations for this tumor subtype. In conventional triple-negative breast cancer, systemic chemotherapy is a cornerstone of treatment. FLMC, however, is biologically unique; its low risk of metastases makes routine chemotherapy harder to justify when no other high-risk features are present. A consensus review by the European Working Group for Breast Screening Pathology concluded that available data do not support the benefit of adjuvant chemotherapy in reducing the risk of local recurrence or metastasis, and that many patients with FLMC are unlikely to benefit from systemic treatment [5]. The disease-free status of our patient at two years of follow-up supports the appropriateness of surgery and radiation alone in carefully selected cases. Nonetheless, extended surveillance is warranted given the tumor's propensity for local recurrence and its documented, albeit rare, capacity for aggressive behavior.

## Conclusions

FLMC is a rare and diagnostically challenging subtype of MBC that closely resembles desmoid-type fibromatosis histologically. Accurate diagnosis requires a high index of suspicion, adequate tissue sampling, and a comprehensive IHC panel. While the absence of nuclear β-catenin staining is an important feature for distinguishing FLMC from desmoid-type fibromatosis, it is not independently diagnostic. Confirmation of positive epithelial differentiation through keratin immunostaining is the essential finding, and β-catenin negativity should always be interpreted in the context of the complete morphologic and immunohistochemical profile. Despite its triple-negative immunophenotype, FLMC behaves unlike conventional triple-negative breast cancer, with low rates of regional lymph node and distant metastases in most cases. Followed by no evidence of disease at 24 months, this case illustrates a breast-conserving approach with adjuvant radiotherapy and omission of chemotherapy as a potentially appropriate strategy in carefully selected patients, though this follow-up interval is relatively short for a tumor with a recognized propensity for local recurrence and longer-term data are needed. Long-term clinical and imaging follow-up remain important given the tumor's recognized propensity for local recurrence and its rare but documented potential for aggressive behavior. Additional case reports and prospective data are needed to expand our understanding of this uncommon tumor and inform future management guidelines.
